# Complete chloroplast genome sequence of *Gynostemma pentaphyllum* (Cucurbitaceae), a perennial medicinal herb

**DOI:** 10.1080/23802359.2019.1688726

**Published:** 2019-11-12

**Authors:** Yancai Shi, Rong Zou, Bingbing Liu

**Affiliations:** a Institute of Loess Plateau, Shanxi University, Taiyuan, Shanxi, China;; b Guangxi Institute of Botany, Guangxi Zhuang Autonomous Region and Chinese Academy of Sciences, Guilin, China

**Keywords:** *Gynostemma*, chloroplast genome, phylogenetic analysis

## Abstract

*Gynostemma pentaphyllum* (Cucurbitaceae) is a perennial medicinal herb widely distributed in China. It’s well known for its medicinal values due to contains important medicinal components gypenosides. Here, we first report and characterize its complete chloroplast genome based on Illumina paired-end sequencing data. The complete plastid genome was 154,457 bp, which contained inverted repeats (IR) of 25,603 bp separated by a large single-copy (LSC) and a small single copy (SSC) of 84,998 bp and 18,253 bp, respectively. The cpDNA contains 132 genes, comprising 81 protein-coding genes, 37 tRNA genes, 8 rRNA genes and six processed pseudogenes. The overall GC content of the plastome is 37.1%. The phylogenetic analysis of 17 selected chloroplast genomes demonstrated that *G. pentaphyllum* is closely related to the congeneric *G*. *compressum*.

*Gynostemma pentaphyllum* (Thunb.) Makino, which belongs to the *Gynostemma* genus in Cucurbitaceae family, is a perennial medicinal herb widely distributed in China. It’s a traditional oriental medicinal herb used as tea since ancient time. It contains important medicinal components called gypenosides, which are reportedly effective in the treatment of many illnesses, such as inflammation, cardiovascular diseases, and cancer (Bai et al. 2010). However, genetic and genomic resource of the species is very limited. Herein, we first report and characterize its complete plastome based on Illumina paired-end sequencing data, which will contribute to the further studies on its genetic research and resource utilization. The annotated cp genome of *G. pentaphyllum* has been deposited into GenBank with the accession number MN583314.

In this study, *G. pentaphyllum* was sampled from in Guangxi Zhuang Autonomous Region of China, located at 110°04′26″ E, 25°42′11″ N. A voucher specimen (Y.-C. Shi et al. H145) was deposited in the Guangxi Key Laboratory of Plant Conservation and Restoration Ecology in Karst Terrain, Guangxi Institute of Botany, Guangxi Zhuang Autonomous Region and Chinese Academy of Sciences, Guilin, China. The experiment procedure is as reported in Zhang et al. (2019). Around 2 Gb clean data were used for the cp genome de novo assembly by the program NOVOPlasty (Dierckxsens et al. 2017) and direct-viewing in Geneious R11 (Biomatters Ltd., Auckland, New Zealand). Annotation was performed with the program Plann (Huang and Cronk 2015) and Sequin (http://www.ncbi.nlm.nih.gov/).

The chloroplast genome of *G. pentaphyllum* is a typical quadripartite structure with a length of 154,457 bp, which contained inverted repeats (IR) of 25,603 bp separated by a large single-copy (LSC) and a small single copy (SSC) of 84,998 bp and 18,253 bp, respectively. The cpDNA contains 132 genes, comprising 81 protein-coding genes, 37 tRNA genes, 8 rRNA genes and six processed pseudogenes. Among the annotated genes, 15 of them contain one intron (*pet*B, *pet*D, *ndh*A, *ndh*B, *rps*16, *rps*12, *rpoC*1, *rpl*16, *rpl*2, *trn*A-UGC, *trn*I-GAU, *trn*G-GCC, *trn*K-UUU, *trn*L-UAA and *trn*V-UAC), and two genes (*clp*P and *ycf*3) contain two introns. The overall GC content of the plastome is 37.1%.

To identify the phylogenetic position of *G. pentaphyllum*, phylogenetic analysis was conducted. A neighbor joining (NJ) tree with 1000 bootstrap replicates was inferred using MEGA version 7 (Kumar et al. 2016) from alignments created by the MAFFT (Katoh and Standley 2013) using plastid genomes of 17 species. It showed the position of *G. pentaphyllum* was close to the congeneric *G*. *compressum* (Figure 1). Our findings can be further used for plastome evolution, population genomic and phylogenomic studies of Cucurbitaceae. It will also provide fundamental data for the utilization and management of this important medicinal plant.

**Figure 1.**
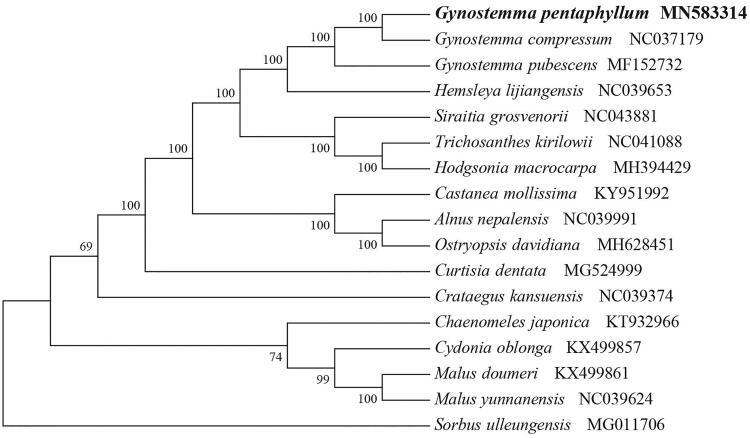
NJ phylogenetic tree of *G. pentaphyllum* with 16 species was constructed by chloroplast plastome sequences. Numbers on the nodes are bootstrap values from 1000 replicates. *Sorbus ulleungensis* was selected as outgroups.
